# The visual familiarity effect on attentional working memory maintenance

**DOI:** 10.3758/s13421-024-01548-1

**Published:** 2024-03-19

**Authors:** Philippe Schneider, Evie Vergauwe, Valérie Camos

**Affiliations:** 1 University of Fribourg, Fribourg, Switzerland; 2 University of Geneva, Geneva, Switzerland

**Keywords:** Working memory, Long-term memory, Refreshing, Visual familiarity

## Abstract

Attentional refreshing has been described as an attention-based, domain-general maintenance mechanism in working memory. It is thought to operate via focusing executive attention on information held in working memory, protecting it from temporal decay and interference. Although attentional refreshing has attracted a lot of research, its functioning is still debated. At least one conception of refreshing supposes that it relies on semantic long-term memory representations to reconstruct working memory traces. Although investigations in the verbal domain found evidence against this hypothesis, a different pattern could emerge in visuospatial working memory in which absence of refreshing evidence has been observed for stimuli with minimal associated long-term knowledge. In a series of four experiments, the current study investigated the hypothesis of an involvement of semantic long-term representations in the functioning of attentional refreshing in the visuospatial domain. Both cognitive and memory load effects have been proposed as indexes of attentional refreshing. Therefore, we investigated the interaction between the effects of visual familiarity (a long-term memory effect) and cognitive load on recall performance (Experiments 1A and 1B), as well as the interaction between the effects of visual familiarity and memory load on the response times in a concurrent processing task (Experiments 2A and 2B). Results were consistent across experiments and go against the hypothesis of the involvement of semantic long-term memory in the functioning of attentional refreshing in visuospatial working memory. As such, this study corroborates the results found in the verbal domain. Implications for attentional refreshing and working memory are discussed.

**Supplementary information:**

The online version contains supplementary material available at 10.3758/s13421-024-01548-1.

Working memory (WM) has been defined as the part of our cognitive system dedicated to the maintenance of a limited amount of information for a short duration as well as the online processing of currently relevant information (Barrouillet & Camos, 2015; Cowan, 2017; Oberauer, 2009). It is one of the most extensively investigated topics in psychological science, as it is central to our day-to-day functioning and has been linked to several higher cognitive functions (Conway et al., 2002, 2007, for a review). One central debate about WM is its relationship to semantic long-term memory (LTM), where a seemingly infinite quantity of semantic information can be kept for long-time storage and retrieval (Bahrick et al., 1975; Brady et al., 2008). On the one hand, some authors argue that semantic LTM and WM are not functionally distinct, but that WM is basically activated semantic LTM (Cowan, 2008; Oberauer, 2002). On the other hand, other authors consider WM as a system separate from semantic LTM (Baddeley, 2000; Baddeley & Hitch, 1974; Barrouillet & Camos, 2015). One possible avenue to investigate the link between WM and semantic LTM would be to explore how semantic LTM factors impact WM maintenance processes. Accordingly, the present study focuses on one maintenance mechanism in particular, called attentional refreshing, which is an attention-based mechanism that maintains mnemonic traces active in WM against decay and interference (see Camos et al., 2018, for a review). Among the different conceptions of refreshing, one suggests that it relies (at least in part) on semantic LTM representations (Barrouillet & Camos, 2015). This conception assumes that attentional refreshing uses information stored in semantic LTM to reconstruct WM traces. Barrouillet and Camos (2015) suggested that more familiar items would benefit from better reconstruction, in other words being more efficiently refreshed, than less familiar ones, leading to an interaction between manipulations of familiarity and refreshing. However, previous examinations of the influence of item familiarity on attentional refreshing efficiency used verbal materials and provided evidence against this hypothesis (Camos et al., 2019; Labaronne et al., 2023). It is nevertheless possible that this finding is specific to verbal WM. The aim of the present study was to extend the examination of the effect of item familiarity on attentional refreshing to the visuospatial domain of WM.

## Attentional refreshing in WM

Attentional refreshing is conceived as a domain-general maintenance process that uses central or executive attention to keep representations active in WM for subsequent processing (Camos et al., 2018; Johnson, 1992). It is thought to work by increasing the activation level of just-presented information, thus prolonging its accessibility in WM. This increase in activation is achieved by focusing attention on the WM representations of just-presented information. Thus, refreshing refers to the act of briefly thinking of WM representations, when the just-presented information is not directly available to our senses anymore. According to the time-based resource sharing model (TBRS model; Barrouillet & Camos, 2015), attentional refreshing cannot be performed at the same time as another attention-demanding process due to a central bottleneck. Thus, in case of competition between attentional refreshing and another attention-based process, attention has to be shared in a time-based manner between the two processes.

This time-based sharing of attention has two consequences, which can be considered as two indexes of the functioning of attentional refreshing. A first index, named the cognitive load effect, refers to the observation that recall performance in a complex-span paradigm, in which the maintenance of information is interspersed with concurrent processing, is linearly correlated with the cognitive load of the concurrent processing task (e.g., see for review, Barrouillet & Camos, 2015, 2021). The more this concurrent task requires attention per unit of time, the worse is recall performance. In the TBRS model, the cognitive load reflects the proportion of time during which attention is dedicated to concurrent processing and thus unavailable for maintenance purposes through attentional refreshing. Accordingly, a more attention-demanding concurrent task would divert general attention from refreshing the to-be recalled items for longer periods of time, compared with a less attention-demanding concurrent task. Hence, the cognitive load of a processing task can be manipulated by varying either the overall time available to process a given number of stimuli of the concurrent task (i.e., the retention interval in complex span task) or the number of stimuli to process in the processing phase of a given duration (Barrouillet et al., 2007; Barrouillet & Camos, 2012). For example, in a complex span task in which participants maintained series of letters while reading series of digits, Barrouillet et al. (2004) reported that the amount of letters participants were able to recall dropped from 4.90 to 3.41 letters on average when the cognitive load of the concurrent task increased from 0.4 to 2 digits per second (with nine different values of cognitive load).

A second index, named the memory load effect, is the reciprocal effect of the previous one. According to the TBRS model, when attentional refreshing takes place, attention is not available for concurrent processing. As a result, refreshing postpones the execution of concurrent processing. When more WM representations have to be refreshed in a sequential manner, the postponement becomes longer, which is reflected in longer response times to a concurrent processing task. Several studies showed indeed that the mean response time to a concurrent task increases for each additional item to be maintained in WM. For example, Vergauwe et al. (2014) developed a paradigm inspired by the Brown–Peterson task (Brown, 1958; Peterson & Peterson, 1959). This task was similar to a complex span task, with the difference that item presentation was not interspersed by a concurrent processing task. Instead, the processing task lasted for a fixed duration between item presentation and recall. The authors reported that the response times to the concurrent tasks increased linearly with the number of memory items (see Camos et al., 2019, for similar findings). Jarrold et al. (2011) showed a similar pattern of results using a complex-span task where they analyzed the response times to the concurrent task following each image presentation. In these studies, response times to the concurrent task were approximately 40 ms slower for each additional item held in WM. Since refreshing and concurrent processing cannot be done simultaneously, the processing of the concurrent task is postponed until all the items held in WM are refreshed, and the 40-ms slope found in the aforementioned studies is then interpreted as the time our cognitive system takes to refresh one memory item.

Despite the extensive research on attentional refreshing, its inner working is still up to debate. Researchers agree that central attention is needed for refreshing (e.g., Vergauwe et al., 2012), but otherwise several different proposals about its functioning have been put forward. On the one hand, its functioning has been described as a mechanism that uses semantic LTM representations to reconstruct WM representation (akin to a redintegration process; Barrouillet & Camos, 2015; Thorn et al., 2005). On the other hand, Vergauwe and Cowan (2015) described its functioning as a rapid scanning of the current content of WM. The main difference between the two proposals lies in the involvement of semantic LTM knowledge. Thus, investigating to what extent semantic LTM factors can influence attentional refreshing could give us information on the relationship between semantic LTM and WM, which coincidentally could inform on the functioning of attentional refreshing itself.

## The role of semantic LTM in attentional refreshing

As said earlier, some proposals on the functioning of attentional refreshing propose that the mechanism relies on semantic LTM representations to support the maintenance of information in WM. One way to test this hypothesis is to examine how attentional refreshing is affected by well-replicated effects showing an effect of semantic LTM factors on WM functioning. To that end, two effects have been examined in relation to attentional refreshing in verbal WM (Camos et al., 2019): the lexicality effect and the lexical frequency effect. The lexicality effect is the observation that words are better recalled than nonwords. The lexical frequency effect is the observation that more frequent words are better recalled than less frequent words. Although these effects have been replicated several times in WM tasks (e.g., Hulme, et al., 1991; Majerus & Van der Linden, 2003), its locus in WM is still up to debate. One possibility is that these effects reflect a differential impact of attentional refreshing that depends on item familiarity, such that more frequent words are refreshed more efficiently than less frequent words, because their representation in semantic LTM is better defined or more easily accessible. To test this hypothesis, a recent study manipulated the cognitive load orthogonally to either the lexical frequency of words or the lexicality of memory items, in a complex span task (Camos et al., 2019). In a first experiment, series of low-frequency or high-frequency words were presented in a complex-span task in which the cognitive load of the concurrent task was also manipulated. The authors’ rationale was as follows: If more frequent words are refreshed more efficiently and thus better protected from forgetting than less frequent words, then the lexical frequency effect (the difference in recall performance between the low- and high- frequency words) should be larger in trials where participants had more time to refresh (low cognitive load trials) than in trials where they had less time to refresh (high cognitive load trials). When there is less time to engage in attentional refreshing, the difference between high- and low-frequency words would be smaller, as the advantage for more frequent word would have less opportunity to manifest. If so, this should result in a statistical interaction between the lexical frequency of memory words and the cognitive load of the concurrent task. Results showed evidence against this interaction of interest, contradicting the aforementioned hypothesis. A similar absence of interaction with cognitive load was observed in a second experiment in which the lexicality of the memoranda (words vs. nonwords) was manipulated. In two additional experiments, Camos et al. (2019) tested the same hypothesis but used another index of attentional refreshing (i.e., the effect of memory load on response times in a concurrent processing task). In particular, Experiment 3 uses low- and high-frequency words as memoranda in a Brown–Peterson task, following the same logic as the task developed by Vergauwe et al. (2014). The idea was that if the memory load effect on the response times of the concurrent task indexes attentional refreshing and if high-frequency words are refreshed more efficiently than low-frequency words, then an interaction should be detected between both variables. Specifically, it was expected that high-frequency words would postpone the concurrent task to a lesser extent than low-frequency words, because high-frequency words would be refreshed more quickly. The results contradicted this prediction, showing evidence against the aforementioned interaction. This pattern of results was replicated in a final experiment, in which the lexicality of memory items was manipulated instead of the lexical frequency in the same Brown–Peterson task. Together, the four experiments reported in Camos et al. (2019) provided evidence against an interaction between semantic LTM factors and attentional refreshing, which casts doubt on the hypothesis of an involvement of semantic LTM in the functioning of attentional refreshing.

Other studies investigating attentional refreshing reached similar conclusions using different manipulations (Labaronne et al., 2023; Loaiza & Camos, 2018; Rosselet-Jordan et al., 2022). For example, Labaronne et al. (2023) manipulated the lexicality of the memoranda in a complex span task in which the cognitive load of a concurrent parity task was varied. As in Camos et al. (2019), the authors expected an interaction between the cognitive load effect and the lexicality of the memory items, with a cognitive load effect in words but not for pseudowords. However, contrary to this prediction, the cognitive load effect impaired recall performance in both words and pseudowords. Although this finding confirmed Camos et al.’s (2019) results, the absence of an implication of semantic LTM on refreshing while overall recall performance was affected by LTM effects left open the question on the locus of the implication of semantic LTM on WM. Moreover, despite the fact that attentional refreshing is conceived as a domain-general mechanism that can be used for both verbal and visuo-spatial memory materials (Barrouillet & Camos, 2015; Cowan, 1995), all the experiments described above were limited to the verbal domain. Given that attentional refreshing has been described as a domain-general mechanism (Barrouillet & Camos, 2015; Cowan, 1995), one could assume that it should operate similarly for verbal and visuospatial domains, but some models of WM, such as the multicomponent model (Baddeley et al., 2021) propose that visuo-spatial and verbal information are processed by distinct mechanisms in WM.

## Differences between verbal and visuospatial domains in WM

The most influential model of WM assumes a functional separation between verbal and visuospatial WM (Baddeley, 1986, 2000; Baddeley & Logie, 1999). It could then be that, even though attentional refreshing is deployed in the same way for verbal and visuospatial information, specificities about how information is actually represented could differ between the verbal and visuo-spatial domains and this could, in turn, impact the functioning of attentional refreshing. This possibility is supported by the findings of Ricker and Cowan (2010), which showed that some item features in the visuospatial domain are inevitably forgotten after a retention interval compared with words or letters that are less susceptible to temporal decay. The authors interpreted this pattern of results as possible evidence for a differential impact of attentional refreshing between domains, and that this discrepancy would be due to differences in how information is represented between the verbal and the visuospatial domain.

In addition, several studies reported an absence of cognitive load or memory load effects for different visuospatial memoranda. In one experiment, Vergauwe et al. (2014) found no effect of memory load on the response times to a concurrent task in a Brown–Peterson paradigm where participants had to maintain distinct fonts of the same letter. In the same vein, Ricker and Vergauwe (2020) consistently failed to find an effect of cognitive load manipulation in a Brown–Peterson task in which participants had to maintain the location of a dot on the edge of a circle. In all their experiments, the authors failed to find an effect of an attentional refreshing manipulation on specific visuospatial items, and argued that this discrepancy with the literature could be due to the fact that the visuospatial memoranda used in these experiments lack stable semantic LTM representation, rendering them impossible to refresh. These findings suggest that the visuospatial domain differs from the verbal domain, in which the cognitive and memory load effects have been consistently observed whatever the nature of the verbal memory items (see Barrouillet & Camos, 2015, for review). Moreover, the authors’ suggestion to explain the lack of cognitive and memory load effects on these visuospatial memoranda implicitly acknowledges that the functioning of attentional refreshing relies, at least in part, on semantic LTM. This is at least partially congruent with Barrouillet and Camos’s (2015) proposal on how attentional refreshing uses information stored in semantic LTM to reconstruct degraded memory traces. Hence, the visuospatial domain may have some specificities such that visuospatial items can be refreshed only if a stable semantic LTM representation exists for these items. The current study aimed at directly testing this proposal by manipulating the quality of LTM representations through the familiarity effect in visuospatial WM, and by testing to what extent indexes related to refreshing are affected by these manipulations.

## The current study

The aim of this series of experiments was to investigate whether semantic LTM factors influence attentional refreshing of information in visuospatial WM. Throughout four experiments, we adopted the approach of Camos and colleagues (2019) but in the visuospatial domain. Hence, we needed an equivalent of lexical frequency or lexicality manipulation implemented by Camos et al. (2019) for the visuospatial domain.

**Fig. 1:**
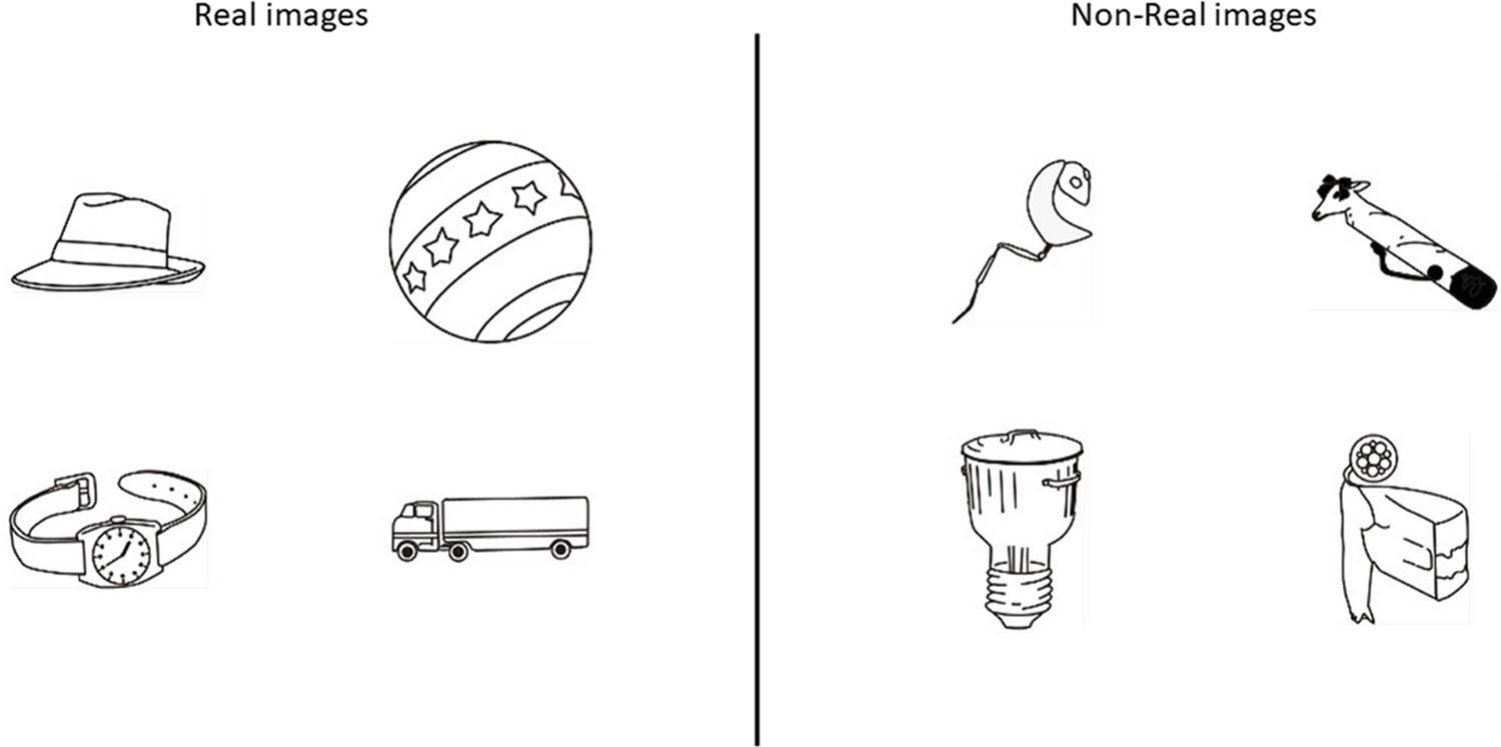
Examples of images used in the study

The lexical frequency effect relies on the fact that we do not encounter all words at the same frequency in our day-to-day life. By definition, items are more familiar when we encounter them more frequently. It has been thus hypothesized that the strength of a trace in semantic LTM depends on how frequently we have encountered the information before having to maintain it. To emulate this in the visuospatial domain, we used the visuospatial familiarity effect by creating two pools of images that vary in their degree of familiarity. The high-familiarity images (here after the “real” images) were black-and-white drawings of real-life objects taken from the Snodgrass and Vanderwart (1980) image set, while the low-familiarity images (here after the “nonreal” images) were taken from Soldan et al. (2008), who created images by smoothly put together features from different images taken from the Snodgrass and Vanderwart image set (see Fig. 1). The nonreal images were then approximately as complex as the real images but were not representing day-to-day objects, a difference similar to the one between words and pseudo-words.

We used the images of real vs- nonreal objects in two series of two experiments. In Experiments 1A and 1B, we used a complex span task in which we manipulated orthogonally the familiarity of the images to maintain and the cognitive load of the concurrent task in a within-subject design; Experiment 1B implementing a stronger manipulation of the cognitive load than Experiment 1A. The aim was to investigate whether recall performance was better for high-familiarity images compared with low-familiarity ones and, most importantly, whether the cognitive load effect would interact with the image familiarity. We expected that, if attentional refreshing is facilitated by a greater accessibility to semantic LTM representations, then low-familiarity images would yield a smaller (or even no) cognitive load effect than high-familiarity images, because the former would benefit less from refreshing opportunities than the latter.

In Experiments 2A and 2B, we implemented a slightly different complex span task, designed to investigate how the response times to the concurrent task are modulated by the image familiarity as well as by the number of images to maintain. In particular, we expected that, if the real images are refreshed more efficiently than the nonreal images, then the memory load effect on response times should be more pronounced for the nonreal images than for the real ones, which would result in a statistical interaction between memory load and image familiarity. If low-familiarity images take longer to refresh, then the resulting postponement of concurrent processing caused by each additional memory item should be more important for low-familiarity images, relative to high-familiarity images. Contrary to Camos et al. (2019), we choose to use the complex-span paradigm instead of the Brown–Peterson paradigm to evaluate response times to the concurrent task in regard to the memory load, because the complex span paradigm gives a higher number of data points per experimental cell during one session than the Brown–Peterson task, while not lengthening the overall duration of the experiment. This is due to the fact that, in the Brown–Peterson paradigm, only one memory load condition is presented per trial. By contrast, response times to the concurrent task can be sampled after the presentation of each additional memory image in the complex-span task and thus, one trial of the complex-span paradigm induces several memory loads along the trial. Since our theoretical question focused on the presence or the absence of statistical interactions, we used Bayesian statistics throughout our analyses, as it can give evidence for the presence or the absence of an effect, contrary to the frequentist statistical approach.

## Experiment 1A

In Experiment 1A, participants had to maintain the real and nonreal images described earlier while performing a parity judgment task. We manipulated orthogonally the familiarity of images to maintain and the cognitive load of the concurrent task. We selected the parity judgment task as concurrent task because it requires participants to make a response selection and ensured that general attention is involved. This task is also known for impacting attentional refreshing (e.g., Barrouillet et al., 2007). To maximize the cognitive load difference between conditions, a fixed number of digits appeared slowly in the low cognitive load condition (one digit every 2,000 ms) and at twice this pace in the high cognitive load condition (one digit every 1,000 ms), which reduces the availability of attention for maintenance purposes. Participants were also under articulatory suppression during image presentation and the concurrent parity judgment task, to minimize the possibility to recode and rehearse the presented information verbally. This was particularly important as it can be assumed that the real images are easier to recode into verbal code than the nonreal images. We predicted that, if high-familiarity images are refreshed more efficiently than low-familiarity images, then the cognitive load effect should be larger in the high-familiarity items than in the low-familiarity items, because the former would benefit more from the availability of attention for refreshing during the processing phase.

### Method

#### Participants

Forty students from the University of Fribourg and Geneva (38 women, mean age = 20.8± 2.0 years) participated in this experiment. Participants had normal or corrected-to-normal vision. They were compensated with partial course credits or cinema tickets. Every participant read and signed an informed consent form. Ethical approval was given by IRB of both the University of Fribourg and University of Geneva.

#### Material

The memory items for the high-familiarity condition consisted of 84 images of real objects taken from the Snodgrass and Vanderwart (1980) database. We selected images related to concepts with high lexical frequency nouns (mean frequency = 107.9 ± 136.3 per million, range: 20–788, taken from the Lexique3 French words database; New et al., 2001, 2004) to ensure that the images represented well known objects. For example, images could represent an airplane, a finger, a pan (see Fig. 1). The stimuli for the low-familiarity condition comprised 84 nonreal black and white images that were taken from Soldan et al. (2008). Each image was composed of features coming from different images from the Snodgrass and Vanderwart’s image set fused together to create nonsensical objects (see Fig. 1). The complete set of images can be found on the OSF page related to this study (https://osf.io/xdawz).

#### Procedure

**Fig. 2:**
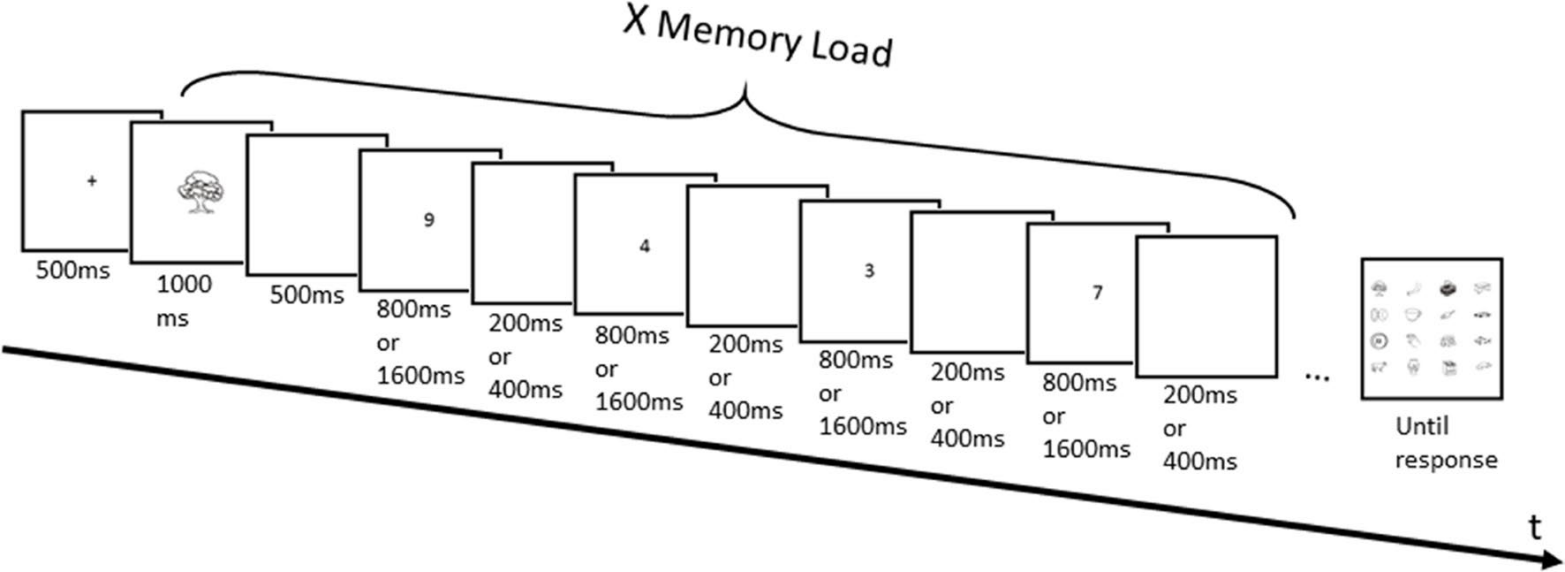
Illustration of the successive events in a complex-span task trial in Experiment 1A. The presentation of a memory image and the phase of parity judgments are repeated for each image presented in the trial

This experiment used a complex span paradigm (see Fig. 2) in which stimuli to remember were the real and the nonreal images described earlier. The concurrent task was a parity judgment task with digits ranging from 1 to 9, 5 being excluded to have the same number of odd and even digits. The experiment was divided in two blocks, one for the real images and the other for the nonreal images. The order of the blocks was counterbalanced across participants. Each block consisted of a maximum of 42 trials. An increasing length procedure was used, from 1 to 7 memory items (length 1 to 7). Participants were informed of the change of lengths by a screen mentioning the new length. Each block started with six Length 1 trials, three in each cognitive load condition, the order of which was randomized for each participant. We implemented a stop rule in each block. If a participant recalled all images in the correct order in at least one trial from Length 1, the six trials from the next length (Length 2) were presented, and so on. In case of no successful trial for a given list length, the block would stop and the prompt for the next block would appear (or the experiment would stop if this was the last block).

Participants were seated at approximately 40 cm from the screen. At the start of every trial, the pace of the concurrent task was given: “rapide” or “lent” (*fast* and *slow* in French, respectively) and would remain on screen until the participant pressed the space bar to start the trial. Then, a fixation cross appeared in the center of the screen for 500 ms, followed by the first image to memorize, which stayed on screen for 1 s (see Fig. 2). The image was presented in the center of the screen and measured approximately 10 cm × 6 cm. After the image presentation and a 500-ms blank screen, the parity judgment task started. Participant had to decide whether four sequentially presented digits were odd or even by pressing the left or right mouse button, respectively. In the high cognitive load condition, digits appeared for 800 ms with a 200-ms blank screen after each digit. In the low cognitive load condition, digits stayed on screen for 1,600 ms with a 400-ms blank screen. The parity judgment phase lasted then 8 s and 4 s in the low and high cognitive load conditions, respectively. The digits were randomly selected without replacement for each parity judgment task phase. At the end of a trial, participants were presented with a total of 16 images on screen. These included the target images from the trial, as well as nontarget images randomly selected from the image pool of the current block. Importantly, the nontarget images were not allowed to be targets in the preceding or following trial. Participants had to click on the images that were presented in the order of presentation, using the left mouse button. We choose an array of 16 images because it was a good balance between having a low chance level without the images being too small on screen during the response phase. Participants had to click on the same number of images as the length of the trial and could not correct themselves after they had clicked on an image. They had no time limit to answer. Since there were always 16 images presented simultaneously during the response phase, the number of nontarget images was always equal to 16 minus the number of presented items in the current trial. In the response phase, each image was of the size 3 cm × 2 cm, and they were presented on a 4 × 4 matrix centered in the middle of the screen. Each image was separated by 2 cm horizontally and vertically to any adjacent images. After participant had given their answer, the screen provided information for the next trial. The choice of images in each trial was randomized for each participant, with the constraint that, within a given block, each image appeared twice as memory item (once in each cognitive load condition) and six times as nontarget during the response phase.

To minimize verbal recoding and the use of subvocal rehearsal, participants had to say “Ba-Bi-Boo” out loud from the start of each trial (i.e., when they pressed space bar to launch the trial) until the response phase, where they could stop. They had to start again at the beginning of the next trial. “Ba-Bi-Boo” was written on the top of the screen from the beginning of the trial until the response phase to remind them to repeat it. Before the experimental trials, participants had four training trials, two of Length 2 and Length 3, with one for each cognitive load for each length. Both image types were used for each list length during these training trials. The images used in the training trials were not used in the experimental trials. Before the training trials, participants received training in the parity judgment task, and had to sort 12 digits in each cognitive load condition, starting from the low cognitive load. The experimenter stayed with the participant during the training phase, but left the participant alone during the experimental trials while monitoring the compliance of the articulatory suppression across the door.

### Results

All analyses in the present study followed a Bayesian statistical approach. In this approach, the resulting statistic is a number comprised between 0 and positive infinity. This number can be interpreted straightforwardly as how many times a model explains the data better (or worse) than another model. For example, a model with a Bayes factor of 10 when compared with the null model implies that this model explains the data 10 times better than the null model. The Bayes factor can be calculated at the model level (as we just mentioned) or at the variable level. When calculated at the variable level, it is called the Bayes factor for the inclusion (or exclusion) of a factor, which is the Bayes factor of the models with the variable of interest divided by the Bayes factor of the models without the variable of interest as predictive variable or in an interaction term (i.e., matched models only). For example, in a 2 × 2 design, with Factors A and B manipulated orthogonally, the Bayes factor for the inclusion of Factor A would be calculated by dividing the Bayes factor for the model containing A + B as predictive factors by the Bayes factor of the model containing B only. If we wanted to evaluate the Bayes factor for the inclusion of the interaction term, we would divide the Bayes factor for the full model (containing A + B + A:B, where A:B represent the interaction term) with the Bayes factor of the model without the interaction (the model containing A + B only). The Bayes factor for (or against) the inclusion of a variable can also be interpreted straightforwardly as how much evidence there is in the data to include (or exclude) the variable in question. All analyses in this paper were performed with the BayesFactor package (Morey & Rouder, 2018) in R (R Core Team, 2020), with default settings. The raw data can be found on OSF (https://osf.io/xdawz).

First, we assessed participants’ performance to the parity judgment task to ensure that participants followed the instructions during the task. We computed the rate of correct parity judgments across the whole experiment. Participants with less than 70% of correct responses across all trials were discarded from analysis. This led to the discarding of data from three participants. We then used this ratio of correct parity judgments as dependent variable in a 2 (image familiarity: real vs. nonreal images) × 2 (cognitive load: high vs. low) Bayesian repeated-measures analysis of variance (ANOVA), with the subject number as the repeated-measure aggregator and its prior was set as “nuisance.” We used the default prior (medium) for the other factors. The best model included only the main effect of cognitive load (BF_10_ = 1.1×10^33^). There was evidence against an effect of the image familiarity (BF_exclusion_= 4.0) and against the interaction between image familiarity and cognitive load (BF_exclusion_ = 3.2). Although processing accuracy was better in the low cognitive load (94% ± 3%) compared with the high cognitive load condition (83% ± 6%), performance was high overall, ensuring participants followed the instructions.

**Fig. 3:**
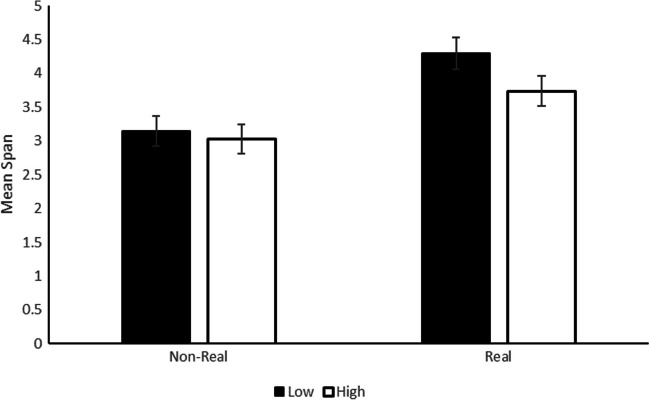
Mean span in Experiment 1A as a function of the type of memory images (on-real or real) and the cognitive load of the concurrent task (low or high). Error bars correspond to standard error

We then calculated the span for each participant in each experimental cell. The span was calculated as follows: each trial in which all images were correctly recalled in the correct position yielded 1 point, thus, as soon as one error was made, the trial yielded 0 points. Then, for each experimental cell, points were summed and divided by the number of trials per list-length. We used this mean span as our dependent variable in a 2 (image familiarity) × 2 (cognitive load) Bayesian repeated-measures ANOVA. As in every analysis in this article, the priors for the effects of interest were set as “medium” (default) and as “nuisance” for the within-subject aggregator. The best model was the one with only the two main effects of image familiarity and cognitive load with a BF10 = 6.8×10^6^ (see Fig. 3). We found very strong evidence for the image familiarity effect, with a lower mean span of 3.1 (±1.4) for the nonreal images than for the real images (mean = 4.0 ±1.4; BF_inclusion_ = 6.1×10^6^). Although in the expected direction, the evidence for an effect of the cognitive load manipulation was more ambiguous, with a BF_inclusion_ of 2.3 in favor of a difference between the low (mean = 3.7 ±1.5) and the high (mean = 3.4 ±1.4) cognitive load conditions. This probably reflected the fact that, when the cognitive load effect was analyzed separately for real and nonreal images using a repeated-measures Bayesian ANOVA, evidence for the cognitive load effect was clear in real images (BF_10_ = 12.5) while evidence supported its absence in nonreal images (BF_01_ = 3.1). Nevertheless, and contrary to what these latter effects may suggest, there was ambiguous evidence against the interaction between cognitive load and image familiarity, BF_exclusion_ = 1.7.

### Discussion

In this first experiment, we observed a strong effect of image familiarity effect on memory performance in a WM task, where real images yield better memory performance than nonreal images. We also found ambiguous evidence against the interaction between image familiarity and cognitive load effect. Even though the cognitive load effect was included in the best model of our memory data, the evidence for the inclusion of the cognitive load effect was rather weak. It is possible that the cognitive load manipulation we implemented was not effective enough to really impact recall performance. Hence, before drawing further conclusions on these findings, we implemented at a stronger cognitive load manipulation in Experiment 1B.

## Experiment 1B

Experiment 1B used the same paradigm as in Experiment 1A, with a few changes. First, the cognitive load manipulation was strengthened. Although we kept the same pace as in Experiment 1A for the low and high cognitive load, eight digits were presented in the high cognitive load condition and only four in the low cognitive load condition. This equalized the total duration (8 s) of the parity judgment task across cognitive load conditions. This way, we managed to manipulate the cognitive load of the secondary task without changing the total duration of each secondary task phase. We also divided each pool of images (real and nonreal) into two sublists. Each participant was attributed one sublist to the high cognitive load condition and the other to the low cognitive load condition, the attribution of which was counterbalanced across participant. This allowed us to use each image only once as a target throughout the whole experiment, minimizing possible training effect on specific images, and also evaluate if one half of the images yielded better performance compared with the other half.

### Method

#### Participants

Forty-one students from the University of Geneva (35 women, mean age: 22.2±6.5 years) participated in this experiment. They were compensated with partial course credits. Every participant read and signed a form of consent. Ethical approval was given by IRB of the University of Geneva. None of them participated in Experiment 1A. One participant was excluded from the analysis because they did not follow the instructions.

#### Material

The same 84 images per image type as in Experiment 1A were used, and divided into two sublists for each type of images. For each participant, one sublist was presented in the low cognitive load condition and the other in the high cognitive load condition. The association between sublist and cognitive load condition was counterbalanced across participants. This allowed us to use every image only once as a target and approximately 3 times as nontarget in the response phase.

#### Procedure

This experiment followed the same procedure as in Experiment 1A, with a few exceptions. Each block consisted of 20 trials (instead of 42 in Experiment 1A), presented in an increasing length structure. The list length varied from two to six (instead of one to seven in Experiment 1A), with four trials per each list length and image type, two in each cognitive load condition. The pace of digits presentation in the parity judgment task was the same as in Experiment 1A, but eight digits were presented in the high cognitive load, and four in the low cognitive load condition to increase the difference of cognitive load between the two conditions (note that four digits were presented in both conditions in Experiment 1A).

### Results

As in Experiment 1A, we first assessed performance on the parity judgment task. We did not exclude any participants as they all exhibited more than 70% of correct response (mean = 89% ± 6%). We then analyzed the ratio of correct parity judgment in a 2 (image familiarity) × 2 (cognitive load) repeated-measures Bayesian ANOVA. This analysis yielded the same pattern of results as in Experiment 1A, with the best model including only the main effect of cognitive load (BF_10_ = 4.1×10^27^). There was very strong evidence for an effect of the cognitive load manipulation (BF_inclusion_ = 5.4×10^27^), with better performance in the low cognitive load condition (95% ± 5%) compared with the high cognitive load condition (86% ± 7%), evidence against an effect of image familiarity (BF_exclusion_ = 4.3), and evidence against the interaction (BF_exclusion_ = 5.2).

**Fig. 4:**
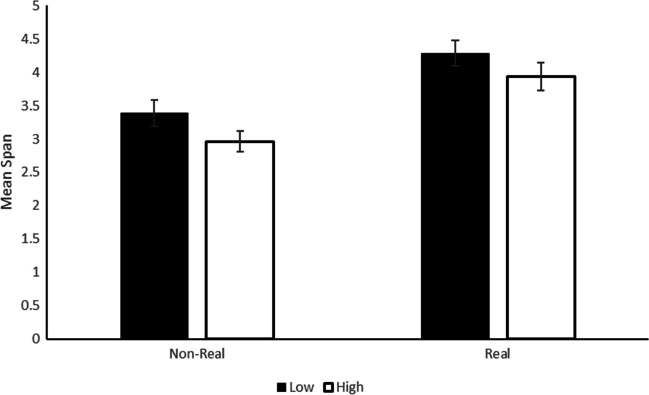
Mean span in Experiment 1B as a function of the type of images to maintain (nonreal or real) and the cognitive load of the concurrent task (low or high). Error bars correspond to standard error

Spans were computed for each participant and each experimental cell in the same way as in Experiment 1A. We first checked the absence of the sublists manipulation on the span score by performing an independent-sample Bayesian *t* test on the span score of participants that had one sublist compared with the other, BF_01_ = 3.1. We then turned to the analysis of the span score as a function of image familiarity and cognitive load manipulation in a 2 (image familiarity) × 2 (cognitive load) repeated-measures Bayesian ANOVA was performed with the calculated span as dependent variable (Fig. 4). The same priors as in Experiment 1A were used for the analysis. The best model included the main effects of image familiarity and cognitive load, BF10 = 1.9×10^10^, with better performance for the real images (mean = 4.1 ± 1.3) than for nonreal images (3.2 ± 1.2, BF_inclusion_ = 3.2×10^9^), and better performance in the low (3.8 ± 1.3) compared with the high cognitive load condition (3.3 ± 1.3, BF_inclusion_ = 19.6). Finally, despite the fact that the strengthening of cognitive load manipulation was successful in impacting recall performance, there was evidence against the predicted interaction between cognitive load and image familiarity, BF_exclusion_ = 4.2.

### Discussion

In this second experiment, we replicated the findings of Experiment 1A but with a clearer outcome. We found more pronounced evidence for the effect of cognitive load as well as for the effect of image familiarity, but also against an interaction between both factors. Overall, Experiment 1B confirmed the absence of the interaction of interest. Under the assumption that the cognitive load effect assesses the operation of attentional refreshing, this finding contradicts the hypothesis that attentional refreshing is supported by semantic LTM representations.

However, to ensure that our conclusion on the absence of an effect of semantic LTM on attentional refreshing was not limited to a single index of attentional refreshing, we extended our investigation of the image familiarity effect to another index of attentional refreshing—namely, the memory load effect on response times in the concurrent task. As explained earlier, several studies showed that an increase in memory load induced a postponement of the response times to the concurrent task, which is interpreted as evidence for attentional refreshing. The use of response times allowed us to examine the effect of image familiarity on attentional refreshing in a more fine-grained measure than span. If the high-familiarity images are refreshed more efficiently than low-familiarity ones, we should expect a difference in the memory load effect on response times as a function of image familiarity. The high-familiarity images should elicit less postponement of the concurrent task than low-familiarity images, that is a smaller memory load effect. Although Experiments 1A and 1B were not designed to directly test this hypothesis, analysis of RT postponement as a function of memory load in these two experiments showed that each new item held in working memory induced a postponement of the reaction times to the concurrent task, as predicted by this hypothesis (see Supplementary Material 1). To test this hypothesis more directly, we adapted the complex-span paradigm used in Experiments 1A and 1B to investigate the impact of memory load on the concurrent task reaction time in two additional experiments.

## Experiments 2A and 2B

The aim of these two experiments was to investigate the effect of image familiarity and memory load on the response times to the parity judgment task in a complex-span paradigm. The hypothesis was that, if the functioning of attentional refreshing is influenced by image familiarity, then we should detect an interaction between the memory load manipulation and the image familiarity manipulation on response times in parity judgments. Experiments 2A and 2B followed the same structure as Experiments 1A and 1B, with the same images to manipulate image familiarity. The only difference between Experiments 2A and 2B concerned the implementation of the concurrent task.

It is important to understand that to examine the memory load effect on response times, participants need to recall correctly the memory items to assure the items were indeed maintained in WM, but participants also have to make correct judgments in the concurrent task. Hence, the way that the concurrent task is implemented can have an impact on participants’ performance. We chose then to administer two variations of the same parity judgment task to check that our findings were not dependent on methodological choices. In Experiment 2A, participants had to judge the parity of a fixed number of digits (4 digits after each memory item), with no time limit on each parity judgment, while in Experiment 2B, the concurrent task between the presentation of memory items lasted for a fixed duration (5 seconds after each memory item), and participants had to judge as many digits as possible during this time. If the memory load effect on response times is larger in the low-familiarity condition compared with the high-familiarity one, this would indicate that low-familiarity images are refreshed more slowly and less efficiently than high-familiarity images.

### Method

#### Participants

In total, 57 participants were recruited and randomly assigned to one of the two experiments, 29 to Experiment 2A (26 women, mean age = 20.8 ± 2.1 years) and 28 to Experiment 2B (26 women, mean age = 21.6 ± 2.4 years). All were students from the University of Fribourg and compensated with partial course credits or cinema tickets. Every participant read and signed a form of consent. Ethical approval was given by IRB of the University of Fribourg. None had participated in Experiments 1A or 1B. One participant from Experiment 2B was excluded from the analysis for having stopped the articulatory suppression.

#### Material and procedure

The same general procedure as in Experiments 1A and 1B was applied, with changes related to the implementation of the concurrent task, the total number of trials, and the memory load manipulation.

First, there were two versions (named A and B) of the parity judgment task, one per experiment. In Experiment 2A, the number of digits to be processed per processing phase was fixed, with four digits presented sequentially. In Experiment 2B, the duration of the parity judgment phase between the presentation of the memory images was fixed, lasting 5 s, and participants had to judge as many digits as possible. Participants were instructed to perform the parity judgment task in such a way that, though aiming at responding as fast and as accurately as possible, they remembered all the memory items in their order of presentation. In both experiments, the next digit appeared 50 ms after the last one was judged, and the delay between image presentation and the beginning of the concurrent task was shortened to 300 ms. Second, the list length varied from two to four images with trials in each length for each type of images (i.e., 48 experimental trials in total). Seventy-six images were selected in both Snodgrass and Vanderwart (1980) and in Soldan and colleagues (2008) image bases. In Experiments 2A and 2B, we did not implement list length longer than four items, because less than half of participants succeeded in two or more trials with a length of five in Experiments 1A and 1B. All trials were presented in one single block, with the trial order randomized for each participant. Before each trial, the number of memory images was indicated. Items for each trial were selected randomly with the limitation that, across all trials, images were not used more than once as memory item for the same participant.

Before the experimental trials, participants performed four training trials to accustom them to the task, two trials of Length 2 and two trials of Length 3, and both kinds of images were used during training, with one trial for each type of images and each list length. The version of the parity judgment task (A or B) was the same in the training and experimental trials. None of the images used in the training trials were used in the experimental trials.

Three different outcome variables were analyzed: recall performance, the first response time of every parity judgment phase (hereafter: Initial-RTs), and the mean response times to all of the subsequent parity judgments of each parity judgment phase (hereafter: Subsequent-RTs). To examine the effect of the memory load on the response times in the parity judgment task, only trials with correct recall were included in the analyses (see Camos et al., 2019; Vergauwe et al., 2014). We split response times in Initial-RTs and Subsequent-RTs, because response times for the first parity judgment can be contaminated with switching processes and consolidation processes, and thus are typically significantly longer than any subsequent response times (Camos et al., 2019; Jarrold et al., 2011; Vergauwe et al., 2014). Since we sampled the Initial-RTs and Subsequent-RTs as a function of memory load, multiple Initial-RTs and Subsequent-RTs values could be measured during a single trial. For example, in a Length 4 trial, we had four measures for both response times: The Initial-RTs following each image presentation (i.e., RT to the first digit that followed memory item presentation) as well as the mean of the Subsequent-RTs following each image presentation, with memory load increasing for later processing phases in the trial (e.g., response times measured in the processing phase following the presentation of the first to-be-remembered image corresponds to response-times measures under Memory Load 1, whereas response times measured in the processing phase following the presentation of the third to-be-remembered image corresponds to response times measures under Memory Load 3).

### Results

As in Experiments 1A and 1B, we used the ratio of correct parity judgments as exclusion criterion. Since our analysis was based on correct parity judgments, we chose an exclusion threshold higher than in Experiments 1A and 1B. Participants should have more than 80% of correct parity judgments to include their data in the reported analyses. No participants were excluded based on this criterion in Experiment 2A, and one was excluded from Experiment 2B. While participants judged the parity of 4 digits presented after each memory image in Experiment 2A (i.e., experiment with fixed number of digits per processing phase), they judged on average 6.2 ± 1.2 digits (range: 3–10) in Experiment 2B, and there was no difference in the number of processed digits between low- and high-familiarity images, as assessed by a two-sided Bayesian *t* test (BF_01_ = 5.5). In both experiments, participants’ rate of correct parity judgments was high (97%, 96%, and 96% in Exp. 2A, and 95%, 95%, and 94% in Exp. 2B, for Lengths 2 to 4, respectively), indicating they followed the instructions well.

Before analyzing response times to the parity judgment task, we first examined recall performance. Because no stop rule was implemented, recall performance was scored via the partial credit unit (PCU). This index is the mean proportion of images correctly recalled in the correct position in each trial. The resulting score has been shown to be a better index of recall performance than span in WM tasks (Conway et al., 2005). We then applied a two-sided paired Bayesian *t* test on PCU score between the low and high visuospatial familiarity condition. In both experiments, this analysis showed a difference between high- and low-familiarity images: Trials with nonreal images (PCU= 0.61 ± 0.24 and 0.63 ± 0.20 in Experiments 2A and 2B, respectively) yielded worse recall performance than trials with real images (PCU= 0.74 ± 0.19 and 0.78 ± 0.16), BF10 = 333 and 1048, for Experiments 2A and 2B, respectively, replicating the visuospatial familiarity effect in memory performance observed in Experiments 1A and 1B.

Next, we analyzed the response times in the concurrent parity judgment task. To investigate the effect of memory load as well as of visuospatial familiarity on response times, only trials with perfect recall were kept, leading to 49% and 47% of the trials being discarded in Experiments 2A and 2B, respectively. The mean Initial-RTs and mean Subsequent-RTs were then computed for each participant in each of the eight experimental cells (2 image familiarity: real or nonreal × 4 memory load: 1 to 4 images). To ensure reliability of our response time measures, experimental cells in which less than two correct trials were available for computing the mean were discarded. This led to discarding 42 experimental cells out of the 232 (8 × 29 participants) in Experiment 2A (3 from the one-image and the two-image conditions, 12 from the three-image, and 33 from the four-image conditions) and 29 out of the 208 cells (8 × 26 participants) in Experiment 2B (six from the three-image and 23 from the four-image conditions). Mean Initial-RTs and Subsequent-RTs were then analyzed in two separate repeated-measures Bayesian ANOVAs.

Before averaging RTs across the subsequent positions (i.e., across all digits presented within a processing episode, except the first digit), we analyzed the effect of position of digits within a processing episode (see Supplementary Material 2). Results of this analysis showed that, in both experiments, there was no effect of digit position (from Position 2 onward) on response time to the concurrent task. Subsequent-RTs from the same parity phase and from the same participant could thus be pooled together.

**Fig. 5:**
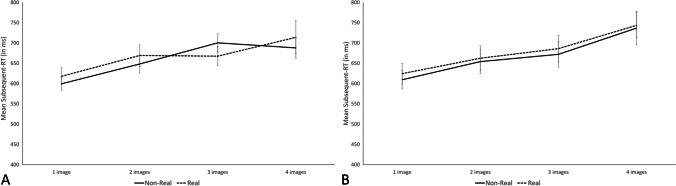
Mean subsequent-RTs (in ms) in Experiments 2A (left) and 2B (right) as a function of the memory load (1 to 4) and the image familiarity (nonreal or real). Error bars correspond to standard error

We thus averaged RTs across subsequent positions (i.e., all except the first position) and analyzed them separately for Experiments 2A and 2B in two 2 (image familiarity: real or nonreal) × 4 (memory load: 1 to 4) repeated-measures Bayesian ANOVAs, one per experiment. Both analyses yielded the same pattern of results (Fig. 5). The best models included only a main effect of the memory load (BF10 = 4.8×10^4^ in Experiment 2A and BF10 = 3.9×10^8^ in Experiment 2B). There was overwhelming evidence for an increase in Subsequent-RTs as a function of the memory load (609 ms ± 101 ms, 659 ms ± 131 ms, 681 ± 122, and 704 ms ± 190 ms for one to four images in Experiment 2A; 617 ms ± 122 ms, 658 ms ± 151 ms, 680 ms ± 163 ms, and 741 ms ± 175 ms in Experiment 2B). We also found evidence against an effect of image familiarity on Subsequent-RTs (652 ms ± 118 ms and 663 ms ± 152 ms for nonreal and real images in Experiment 2A; 655 ms ± 154 ms, and 674 ms ± 156 ms in Experiment 2B), BF_exclusion_ = 5.5 and 5.2 in Experiments 2A and 2B, respectively, and against the interaction between image familiarity and memory load, BF_exclusion_ = 7.2 and 9.7, respectively. To assess the speed of refreshing, we computed the slope of a linear regression on the mean Subsequent-RTs as a function of the memory load, separately for the real and the nonreal images in Experiments 2A and 2B. In Experiment 2A, the linear regression showed an increase of 29 ms per new image held in WM for the real images (*R*^2^ = 0.89) and 32 ms per new image for the nonreal images (*R*^2^ = 0.81). In Experiment 2B, the linear regression showed an increase of 38 ms per new image for the real images (*R*^2^ = 0.97) and an increase of 40 ms per new image for the nonreal images (*R*^2^ = 0.96).

**Fig. 6:**
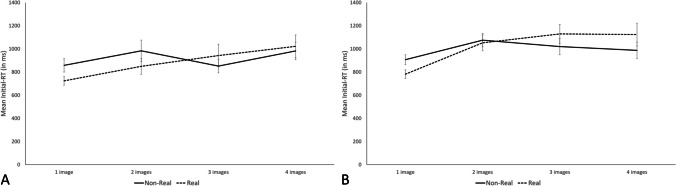
Mean initial-RTs (in ms) in Experiments 2A (left) and 2B (right) as a function of the memory load (1 to 4) and the image familiarity (nonreal or real). Error bars correspond to standard error

The analysis of the Initial-RTs also yielded a similar pattern across the two experiments (Fig 6). In the two experiments, the best model was the full model including both the main effects of memory load and image familiarity as well as the interaction between these two factors (BF10 = 3.8 and BF10 = 1.1×10^10^ in Experiments 2A and 2B, respectively). However, in both experiments, the full model was only ambiguously better than the second-best model that included the effect of the memory load only, with the full model explaining the data only 1.1 and 2.3 times better in Experiments 2A and 2B, respectively. This is likely due to the fact that we found evidence against the inclusion of the type of images in both analyses, with a BF_exclusion_ = 3.1 and 7.0, in Experiments 2A and 2B respectively, but in favor of the inclusion of the interaction (BF_inclusion_ = 4.0 and 13, in Experiments 2A and 2B, respectively). Including image familiarity as a predictor would lower the overall Bayes factor of the models, but this is counteracted by the presence of the interaction. Hence, this probably led to the overall slightly better Bayes factor for the full models compared with the memory-load only models.

To analyze this interaction in more detail, we tested whether the effect of image familiarity was present at each memory load separately. To this aim, we applied dependent two-sided Bayesian *t* test with image familiarity as independent variable on Initial-RTs separately for each memory load. In both experiments, we found convincing evidence for an effect of image type on Initial-RTs for memory load of one (BF_10_ = 25 and 1.1×10^3^, for Experiment 2A and 2B, respectively), but weak evidence at best for a difference between real and nonreal images at higher memory loads (BF_10_ = 2.6, 0.5 and 0.4 for memory load two to four, respectively, in Experiment 2A; BF_10_ = 0.3, 3.2 and 0.8 for memory load two to four, respectively, in Experiment 2B; Fig 6).

### Discussion

Despite differences in the implementation of the concurrent task, both experiments yielded the same pattern of results. First, we replicated the visuospatial familiarity effect in memory performance, nonreal images yielded worse recall performance than real images. Furthermore, as expected, the analysis on the Subsequent-RTs, often considered as an index of the functioning of attentional refreshing, showed that the response times to the concurrent task was influenced by the memory load. Indeed, response times were postponed by approximately 30–40 ms for each additional image to maintain. This is in line with previous findings estimating the speed of refreshing around 40 ms (Camos et al., 2019; Jarrold et al., 2011; Vergauwe et al., 2014), and with the estimations based on the data collected in Experiments 1A and 1B (an increase of 31 ms and 17 ms for real and nonreal images respectively; see Supplementary Material 1). Finally, we also found evidence against the interaction between image familiarity and memory load on the Subsequent-RTs, which, under the assumption that the memory load effect assesses the operation of attentional refreshing, contradicts our hypothesis of an involvement of semantic LTM in attentional refreshing. One could argue that many trials were dropped for the analysis (49% and 47% in Experiment 2A and 2B, respectively), potentially jeopardizing our conclusion. However, this approach is in line with previous studies following the same methodology (Camos et al., 2019; Vergauwe et al., 2014). Theoretically, because we aim to study effects due to WM maintenance, it is important to keep only trials with perfect recall, to ensure that the images were really maintained in WM. To illustrate this point, analysis of subsequent-RTs using all trials in Experiments 2A and 2B regardless of recall performance shows the same pattern of results, but with less steep slopes for the regression analysis of subsequent-RTs on memory load effect (see Supplementary Material 3).

Regarding the results for the Initial-RTs, we found evidence for an effect of memory load on Initial-RTs, with a larger postponement of Initial-RTs induced by higher memory load, but evidence against an effect of image familiarity. However, the image familiarity effect interacted with memory load, with larger Initial-RTs in trials with nonreal images compared with trials with real image for a memory load of one. As Initial-RTs are often considered as indexing consolidation, these findings question the functioning of consolidation process. In the General Discussion, we will elaborate further on this issue.

## General discussion

In a series of four experiments, we investigated the hypothesis that the functioning of attentional refreshing relies on semantic LTM representations in the visuospatial domain of WM. More specifically, we hypothesized that visuospatial items that are more familiar (and thus better represented in semantic LTM) would be refreshed more efficiently and more quickly than less familiar items. In Experiments 1A and 1B, item familiarity and refreshing opportunity were manipulated orthogonally in a complex-span task with a stronger manipulation of refreshing opportunity in Experiment 1B than 1A. In both experiments, we found evidence for an effect of the familiarity manipulation and of refreshing opportunity (although ambiguous in Exp. 1A). However, evidence (ambiguous in Exp. 1A and convincing in Exp. 1B) was gathered against the interaction, which is inconsistent with the hypothesis that attentional refreshing functioning is influenced by semantic LTM factors.

To examine this hypothesis with a more fine-grained measure, Experiments 2A and 2B investigated the effect of familiarity on another index of attentional refreshing: the memory load effect on concurrent task response times. Memory load and the familiarity of visuospatial items were manipulated orthogonally in two complex-span tasks, which differed slightly in their operationalization of the concurrent task. Experiments 2A and 2B yielded the same pattern of results, with evidence for the presence of a memory load effect on response times to the concurrent task, but evidence against an effect of image familiarity and against the predicted interaction, the speed of refreshing thus being similar in high-familiarity and low-familiarity images. Thus, across four experiments, we observed a very consistent pattern of findings. In the following, we will discuss the implications for the functioning of attentional refreshing. We will also discuss the implications regarding the relationship between semantic LTM and WM.

### On the functioning of attentional refreshing

Several accounts of attentional refreshing functioning have been put forward (see Camos et al., 2018, for a review). Although the models differ on several key aspects of attentional refreshing functioning, they tend to agree that attentional refreshing is domain general. Comparing our results to the ones from Camos et al. (2019) lends support to this assumption. Our patterns of results in the visuospatial domain are very similar to the patterns in the verbal domain reported by Camos et al. (2019) in two ways. First, we replicated the cognitive load effect with visuospatial material and a serial recognition paradigm, with worse performance under high cognitive load. This finding extends the results of Camos et al. (2019) that used a written recall procedure. However, the cognitive load effect was less convincing in our experiments, compared with the same manipulation in the verbal domain. Since recognition does not demand a complete reconstruction of the mnemonic traces, less stable WM representations can be recognized relatively easily, whereas active recall of these less stable WM representations may not be possible. Thus, in recognition tasks, it is possible that participants do not actively maintain visuospatial items and just rely on their feeling of familiarity to perform the recognition task. This might be true for Experiment 1A, as the evidence for the cognitive load effect was ambiguous. However, the convincing cognitive load effect in Experiment 1B indicates that, even though we used a recognition procedure, this still elicited active maintenance of the presented items. All in all, the results from Experiments 1A and 1B were in line with previous studies that found a cognitive load effect on recall performance with verbal as well as visuospatial material (Barrouillet et al., 2007; Barrouillet & Camos, 2012; Vergauwe et al., 2010, 2012, 2014).

Secondly, we also extended the memory load effect on concurrent response times to visuospatial material, even though we used the complex-span paradigm instead of the Brown–Peterson task used by Vergauwe et al. (2014) and Camos et al. (2019). A very similar time postponement with increased memory load was observed, with an estimate of the refreshing rate at around 30–50 ms per new item held in WM akin to previous estimates (35–45 ms in Camos et al., 2019; 41 ms in Jarrold et al., 2011; 28–40 ms in Vergauwe et al., 2014). Such a memory load effect is consistent with the idea that attentional refreshing works via a sequential scanning of all items held in WM (Vergauwe & Cowan, 2015). Each item held in WM is refreshed one after the other in a sequential manner, before participants start to process the processing items of the concurrent task. Beside strengthening this account of the functioning of attentional refreshing, our results extend the domain-generality of the memory load effect on the response times to a concurrent task, because the present study observed this effect in a paradigm in which it was never tested before (i.e., complex span task with serial recognition). Because this postponement is considered as an index of attentional refreshing speed, the similarity of slope estimates indicates that visuospatial and verbal items are refreshed at a similar speed. This would be indicative that the same mechanism, probably refreshing, is at play in both domains. Thus, together with the results from Camos et al. (2019), our results reinforce the idea that attentional refreshing is a domain-general mechanism that works in the same way for verbal and visuospatial material.

In addition, our results also show that image familiarity has an impact on recall performance, with conclusive evidence that low-familiarity images yield worse recall performance than high-familiarity images. This difference in performance shows that our manipulation of semantic LTM in the visuospatial domain was effective. Despite the presence of the familiarity effect, we found evidence against the interactions of interest in all four experiments. In Experiments 1A and 1B, the cognitive load effect was similar in both type of images, indicating that low-familiarity images were refreshed as efficiently as high-familiarity ones. This conclusion is supported by the absence of interactions in Experiments 2A and 2B, showing that high-familiarity and low-familiarity images should be considered as being refreshed at the same speed. Taken together, results from our experiments show that image familiarity does not influence the functioning of attentional refreshing, even though it impacts recall performance. This contradicts the hypothesis that attentional refreshing functions via the involvement of semantic LTM.

It is important to note that this conclusion only holds under the assumption that the cognitive load effect on recall and the effect of memory load on Subsequent-RTs reflect the operation of refreshing. Although several researchers have indeed worked under this assumption (e.g., Fanuel et al., 2018; Labaronne et al., 2023; Loaiza & Souza, 2019), one could argue that these effects are reflecting other working memory processes and hence, that our effects do not allow strong conclusions related to refreshing. For the cognitive load effect, at least one alternative explanation has been proposed, whereby the effect would rather reflect the operation of a removal process than of a reactivation process (e.g., Oberauer et al., 2012). Under that assumption, one would rather conclude that the efficiency of removal is not affected by the familiarity of the memory materials (which would make sense, given that the removal process would operate on the processing items, rather than on the memory items). For the memory load effect, a clear alternative explanation is currently not available, because as mentioned, the removal does not operate on memory items, which makes it difficult to understand how their number would affect its efficiency. However, recent research did suggest that the reconfiguration of memory materials may contribute to the effect of memory load on concurrent processing (Joseph & Morey, 2022). Under that assumption, one could conclude that this type of reconfiguration is not affected by the familiarity of the memory materials (although the reconfiguration may explain effects on Initial-RTs, see in the next section). Overall, our data indicate that whatever the nature of the attentional processes taking place during free time in complex span tasks, these processes are not affected by image familiarity and thus, do seem to operate independently from semantic LTM.

### Refreshable and nonrefreshable material

To summarize, our results are congruent with the domain-generality assumption of attentional refreshing and with previous studies that showed an absence of semantic LTM effect on attentional refreshing in verbal WM. However, the question remains as to why some specific visuospatial items are not influenced by attentional refreshing manipulations in some previous studies (e.g., Ricker & Vergauwe, 2020; Schneider et al., 2023; Vergauwe et al., 2014). In their study, Ricker and Vergauwe (2020) failed to find cognitive load effect with some specific visuospatial material (i.e., position around a circle in an angle reproduction task) and put forward several explanations for the absence of this effect. They hypothesized that it could be due to the memory material itself, as an angle reproduction task had never been used before in any study with a cognitive load manipulation. It would suppose that specifics about position around a circle would prevent it from being refreshed. However, this does not seem to hold, as other types of visuospatial memoranda have also yielded an absence of memory load effect (Vergauwe et al., 2014) or seem to not be actively refreshable (Ricker & Cowan, 2010; Schneider et al., 2023). In the current literature, other memoranda for which an effect of attentional refreshing manipulation was not observed are unconventional characters (Ricker & Cowan, 2010), and different fonts of the same letter (Vergauwe et al., 2014). Although the three kinds of memoranda pertain to the same domain, it is not clear how they relate to each other or what the common features are that may drive the similar absence of effect. As suggested by Camos and colleagues (2019), we entertained in the present study the idea that differences in semantic LTM mnemonic traces could be the basis of this discrepancy, but we failed to find support for this hypothesis. Another possible explanation relies on the possibility to verbalize the memoranda. One could argue that fonts of the same letter and unconventional characters are difficult to verbalize without proper training, and that attentional refreshing relies on some sort of verbal recoding. However, this is not congruent with the results from Ricker and Vergauwe (2020). In Experiment 2, they used “canonical” positions around the circle instead of random continuous position, and still found no effect of the cognitive load manipulation, even though the actual position could easily be verbally recoded as “up, down, left or right.”

One last possible explanation relies on the relationship between consolidation and refreshing in WM. Consolidation in WM is a process that transforms fleeting sensory traces into more stable representations in WM (Bayliss, et al., 2015; De Schrijver & Barrouillet, 2017; Ricker & Cowan, 2014; see Ricker et al., 2018, for a review). Ricker and Vergauwe (2020) argued that it could be possible that only well-enough consolidated traces can be refreshed, and that low-level visuospatial features are not easily consolidated. However, recent studies in the verbal domain showed evidence against an interaction between consolidation and refreshing (Bayliss et al., 2015; Labaronne et al., 2023) and against an effect of familiarity on consolidation (Cotton & Ricker, 2021). In our results, we only found reliable differences between familiar and unfamiliar items on Initial-RTs when the memory load was one. Higher memory loads did not show reliable evidence for an effect of image type. If consolidation was influenced by image type, it is rather striking that we found only evidence of such an effect with a memory load of one and not with higher memory load. Thus, it seems our data argue against an impact of familiarity on consolidation process. Regarding the effect of memory load on Initial-RTs, it is rather surprising that the mere consolidation of a newly presented image leads to a memory load effect on Initial-RTs. Indeed, consolidation has been proposed to operate on the memory item that was presented just before, and should therefore not be impacted by the memory load manipulation. It could be that Initials-RTs are also impacted by the refreshing of the already consolidated WM representations (i.e., previously presented images in the same trial), which would be refreshed after the consolidation of the newly-presented memory item and before the response to the first parity judgment. Alternatively, it can be envisioned that the addition of a new image requires a reconfiguration of the output program. This could account for an increase in Initial-RTs throughout serial position, as the output program becoming more and more complex, it needs more time to be reconfigured (see Jones & Macken, 2018; Joseph & Morey, 2022; Myers et al., 2017; Stokes, 2015). Overall, future studies should aim at uncovering, in a more systematic way, which types of material can be influenced by attentional refreshing manipulations and which types cannot, as it could reveal important limitations to the functioning of attentional refreshing.

### Relationships between WM and semantic LTM

Assuming that cognitive load and memory load effects reflect the operation of refreshing, our results go against the idea that semantic LTM is directly involved in the functioning of attentional refreshing. However, since we did find a familiarity effect on recall performance, our results are congruent with the idea that semantic LTM has an impact on WM functioning. The familiarity effect could originate from several WM processes other than those taking place during maintenance period, namely from processes occurring during the encoding and/or the recall phase (see Thorn & Page, 2008, for a review). Regarding encoding processes, it could be that high-familiarity items are encoded more strongly than low-familiarity ones, resulting in better recall performance for high-familiarity items. However, our results in Experiment 2A and 2B on Initial-RTs showed that these response times are only influenced by the memory load and not by image familiarity. This is consistent with the idea that encoding processes happen at the same speed for high-familiarity and low-familiarity items, but does not give information about the strength of this encoding. Finally, Initial-RTs’ pattern of results once again replicates the results from Camos et al. (2019) in the verbal domain. However, we do not consider this as definitive evidence, as Initial-RTs were not our primary variable of interest and the experiments were not designed to directly test the hypothesis that high-familiarity items are better encoded or consolidated than low-familiarity items. Future experiments should aim at investigating this possibility. Alternatively (or in addition to the possible effect at encoding described above), item familiarity could have an impact at the recall stage through the redintegration process (Hulme et al., 1997; Schweickert, 1993). This hypothesis supposes that semantic LTM is used to reconstruct mnemonic traces in WM at recall. Thus, items that are easier to retrieve from semantic LTM (i.e., high-familiarity items) would have a greater probability to be redintegrated than harder-to-retrieve items (i.e., low-familiarity items). To conclude, our findings go against the idea that attentional refreshing of visuo-spatial material relies on semantic LTM. Together with similar findings in the verbal domain (Camos et al., 2019), this tends to support the separation between WM and semantic LTM. Future studies should aim at disentangling the different possible loci for the familiarity effect, and more generally for the LTM effects that impact recall at short term.

## Conclusion

Although we repeatedly found a familiarity effect on recall performance in our study, our data are not congruent with the hypothesis that high-familiarity visuospatial items are refreshed more efficiently than low-familiarity items. This indicates that the familiarity effect in WM does not emerge from the maintenance phase. In four experiments, we found evidence against the hypothesis that attentional refreshing uses semantic LTM to reconstruct traces maintained in WM during the retention interval. The pattern of results corroborates what was already found in the verbal domain. Moreover, our results were inconsistent with the idea that, in the visuospatial domain, information can only be refreshed if a stable semantic LTM traces exist. Future studies should aim at evaluating possible boundary conditions to the functioning of attentional refreshing, an attentional maintenance process that is central to WM.

## Supplementary information

Below is the link to the electronic supplementary material.

Supplementary file1 (DOCX 55 KB)

## Declarations

### Author note

This work was supported by the Swiss National Science Foundation (Grant No. 100019_175960) to V.C. The authors thank Marie Molimard and Anaïs Terrapon for their help in data collection. Although the analysis code is not available, all data and material are available online (https://osf.io/xdawz). The study was not preregistered.
