# Supplementary material for: The visual familiarity effect on attentional working memory maintenance
Source: Mem Cognit. 2024 Mar 19;52(8):1882–99. doi: 10.3758/s13421-024-01548-1 (PMC11588944; doi:10.3758/s13421-024-01548-1)
Supplement: Supplementary file 1 — Supplementary file1 (DOCX 55 KB) [file 13421_2024_1548_MOESM1_ESM.docx]

**Supplementary Material**

1. *Analysis of reaction times to the concurrent task as a function of memory load in Experiments 1A and 1B*

We analysed the reaction times to the parity task as a function of the memory load in Experiments 1A and 1B, separately. Although these two experiments were not designed to investigate whether reaction times to a concurrent task in a complex span are influenced by the memory load, we decided to explore whether such an effect could be found in our first two experiments. To this aim, we first selected only trials in which participants perfectly recalled the memoranda. Then, we computed the mean reaction time to the parity task as a function of the number of images already presented (and this assumed to be held in WM) and the type of images for each participant (i.e., Subsequent-RTs). Then, we used Subsequent-RTs as a dependant variable in a 2 (Image type: real or non-real) X 3 (memory load: 1 to 3) Bayesian repeated measure ANOVA. We only kept memory load from 1 to 3 because these were the only list lengths were more than ¾ of the participants recalled perfectly at least in at least two trials. The best model was the model with only the main effect of the number of images (BF10 = 393), with an increase in RT for each successive presented image. We found evidence against an effect of image type (BF_exclusion_ = 3) and against the interaction (BF_exclusion_ = 4.6). Then, we computed a linear regression on the mean Subsequent-RTs as a function of the number of images. We calculated the slope of this linear regression, which corresponds to the increase in milliseconds for each new image, independently for the real and non-real images. For the real images, the slope showed an increase of 31ms for each new image to maintain (R^2^=0.99). Regarding the non-real images, the slope was 17ms for each new image to maintain (R^2^=0.94). This analysis shows that RT to the concurrent task increases indeed for each new image to be maintained in working memory. However, the value of this slope is to be taken with caution, as the experimental design used to derive these values was not directly created for this task.

1. *Analysis of reaction times to the concurrent task as a function of digit position within a parity phase in Experiments 2A and 2B*

We analyzed the response times (RTs) to the concurrent task as a function of digit position within a parity phase. This included the second, third and fourth digits in Experiment 2A and the second to eighth digits in Experiment 2B^[[1]](#footnote-1)^. A Bayesian ANOVA was performed on RTs to the concurrent task as a function of digit position within a parity phase (2 to 4 in Exp. 2A, and 2 to 8 in Exp. 2B), image type (Real or Non-Real) and list length (2 to 4 memory items). In Experiment 2A, the best model was the null model, with evidence against an effect of digit position (BF_exclusion_ = 4.1), against the interaction between digit position and image type (BF_exclusion_ = 19.3), and against the interaction between digit position and list length (BF_exclusion_ = 1.9). We concluded that digits in position 2 to 4 could be pooled together in Experiment 2A for further analyses.

Experiment 2B showed a different pattern, with the best model including an effect of list length and an effect of digit position (BF10 = 3.0 x 10¹⁹). RTs to the concurrent task decreased with each new digit to judge within a parity phase (651ms ± 158ms, 649ms ± 131ms, 643ms ± 116ms, 620ms ± 101ms, 590ms ± 63ms, 552ms ± 51ms, 535ms ± 69ms, for digit positions 2 to 8 respectively, BF_inclusion_ = 3.5 x 10¹⁷). However, this effect is likely due to the way we pooled the RTs: participants who judged eight digits during a parity phase (limited to 5 s) necessarily did it faster than participants who only sorted 4 or 5 digits. When averaged together, only the response times from “faster” participants (participants that judged more digits) are taken when averaging on the later digit positions. This is evidenced by two analyses: first, when only the digits in positions 2 to 4 were taken into account (maximum number of digits judged by all participants), the repeated Bayesian ANOVA on response time to the concurrent task as a function of digit position within a parity phase (2 to 4 in Exp. 2A and Exp. 2B), image type (Real or Non-Real) and list length (2 to 4 memory items) showed only an effect of list length (BF10 = 1.7 x 10³), with evidence against an effect of digit position (BF_exclusion_ = 32), and evidence against an effect of image type (BF_exclusion_ = 8.7). Secondly, when we analyzed only the trials in which participants were successful in the memory task and managed to judge 8 digits, the same repeated measure Bayesian ANOVA showed evidence against an effect of digit position (BF_exclusion_ = 10.7), against an effect of list length (BF_exclusion_ = 43), and against an effect of image type (BF_exclusion_ = 10.9).

In addition, we also examined whether the Subsequent-RTs differed between Experiments 2A and 2B. To this aim, we applied a 2 (experiment: 2A or 2B) x 2 (image type: real or non-real) x 3 (list length: 2 to 4) x 3 (digit position: 2 to 4) repeated measure Bayesian ANOVA. The best model from this analysis included only the simple effect of list length (BF10 = 1.4 x 10³), with evidence against an effect of digit position (BF_exclusion_ =13.2), against an effect of image type (BF_exclusion_ = 12.8), ambiguous results against an effect of experiment (BF_exclusion_ = 2.8), and evidence for an effect of list length (BF_inclusion_ = 1.2 x 10³). All interaction terms including digit position had evidence against them (all BF_exclusion_ > 9). The effect of list length was due to the fact that Subsequent-RTs were faster with higher memory load, and higher memory load trials (memory load of 3 and 4) where only present in long list-length trials. Since the digit position effect in the first analysis was due to the way we pooled the Subsequent-RTs, RTs for digits in position 2 and onward from the same parity phase and from the same participant were pooled together for further analyses.

1. *Analysis of subsequent-RTs with all trials in Experiments 2A and 2B*

In this supplementary analysis, we aimed to explore the results of Experiments 2A and 2B, when all trials are included regardless of memory performance and compare the results from the analysis of subsequent-RTs as a function of memory load and image familiarity with only correct trials with the same analysis using all trials. This analysis followed the same structure than the one presented in the results section of Experiments 2A and 2B on subsequent-RTs, but all trials were now included. First, we ran two Bayesian repeated measure ANOVAs on subsequent-RTs, for Experiments 2A and 2B separately, as a function of memory load (4 levels: list-length 1 to 4) and image type (2 levels: real or non-real). For both experiments, the best model included only the main effect of memory load (BF_10_ =1.7 x 10^5^ for Experiment 2A; BF_10_ = 1.8 x 10^13^ for Experiment 2B). We then applied a linear regression on the mean subsequent-RTs as a function of memory load to calculate the slope indicating the increase on subsequent-RTs for each new presented image. In Experiment 2A, the regression analysis showed an increase of 25ms (R^2^ = 0.95) for each new high-familiarity image and 18ms (R^2^ = 0.78) increase for each new low-familiarity image. In comparison, the more strict procedure presented in the main text, which found an increase of 29ms (R^2^ = 0.89) for each new high-familiarity images, and 32ms for each new low-familiarity images (R^2^ = 0.81). Regarding Experiment 2B, we found an increase of 24ms (R^2^ = 0.96) for each new high-familiarity image, and 27ms (R^2^ = 0.97) for each new low-familiarity image. In comparison, the more strict procedure found in the main text, which found an increase of 38ms for each new high-familiarity images (R² = 0.97), and an increase of 40ms for each new low-familiarity images (R² = 0.96). Thus, in both Experiments 2A and 2B, the more strict procedure yielded steeper slope than the procedure using all trials. This is likely due to the fact that, by using all trials in the analysis, some trials are included in which participant did not maintain the items, and thus no postponement of processing the main task occurred. This would have resulted in trial with no impact of memory load on processing task, and thus lower (or absent) memory load curve.

1. In Experiment 2B, we included digits up to the eighth position, because 73% of our participants performed correctly at least one trial with a minimum of 8 digits sorted during a single parity phase. [↑](#footnote-ref-1)
